# A low‐cost pipeline for soil microbiome profiling

**DOI:** 10.1002/mbo3.1133

**Published:** 2020-11-22

**Authors:** Anita Bollmann‐Giolai, Michael Giolai, Darren Heavens, Iain Macaulay, Jacob Malone, Matthew D. Clark

**Affiliations:** ^1^ John Innes Centre (JIC Norwich UK; ^2^ Earlham Institute (EI Norwich UK; ^3^ Natural History Museum (NHM London UK; ^4^ University of East Anglia Norwich UK

**Keywords:** amplicon library construction, DNA cleanup, gDNA extraction, microbiome, soil

## Abstract

Common bottlenecks in environmental and crop microbiome studies are the consumable and personnel costs necessary for genomic DNA extraction and sequencing library construction. This is harder for challenging environmental samples such as soil, which is rich in Polymerase Chain Reaction (PCR) inhibitors. To address this, we have established a low‐cost genomic DNA extraction method for soil samples. We also present an Illumina‐compatible 16S and ITS rRNA gene amplicon library preparation workflow that uses common laboratory equipment. We evaluated the performance of our genomic DNA extraction method against two leading commercial soil genomic DNA kits (MoBio PowerSoil® and MP Biomedicals™ FastDNA™ SPIN) and a recently published non‐commercial extraction method by Zou et al. (*PLoS Biology*, 15, e2003916, 2017). Our benchmarking experiment used four different soil types (coniferous, broad‐leafed, and mixed forest plus a standardized cereal crop compost mix) assessing the quality and quantity of the extracted genomic DNA by analyzing sequence variants of 16S V4 and ITS rRNA amplicons. We found that our genomic DNA extraction method compares well to both commercially available genomic DNA extraction kits in DNA quality and quantity. The MoBio PowerSoil® kit, which relies on silica column‐based DNA extraction with extensive washing, delivered the cleanest genomic DNA, for example, best A260:A280 and A260:A230 absorbance ratios. The MP Biomedicals™ FastDNA™ SPIN kit, which uses a large amount of binding material, yielded the most genomic DNA. Our method fits between the two commercial kits, producing both good yields and clean genomic DNA with fragment sizes of approximately 10 kb. Comparative analysis of detected amplicon sequence variants shows that our method correlates well with the two commercial kits. Here, we present a low‐cost genomic DNA extraction method for soil samples that can be coupled to an Illumina‐compatible simple two‐step amplicon library construction workflow for 16S V4 and ITS marker genes. Our method delivers high‐quality genomic DNA at a fraction of the cost of commercial kits and enables cost‐effective, large‐scale amplicon sequencing projects. Notably, our extracted gDNA molecules are long enough to be suitable for downstream techniques such as full gene sequencing or even metagenomics shotgun approaches using long reads (PacBio or Nanopore), 10x Genomics linked reads, and Dovetail genomics.

## Graphical abstract

We present an inexpensive and high‐throughput magnetic particle‐based soil genomic DNA (gDNA) extraction method "SDE" ($0.36) for next‐generation sequencing quality gDNA extraction, scalable to thousands of samples with little hands‐on time. SDE can also be used in combination with our low‐cost amplicon library construction workflow (16S, ITS, $2.5), enabling multiplexing of up to 2304 libraries at a time. Combining SDE and our custom library construction workflow allows utilization of state‐of‐the‐art Illumina sequencing platforms (e.g., NovaSeq) for large‐scale microbiome studies.

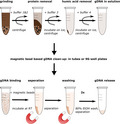

## 1 INTRODUCTION

In the last decade, microbiome studies have been increasing rapidly in popularity, from 4505 publications by December 2010 to 66,250 publications by February 2020 (PubMed reports for search term “microbiome”). Next‐generation sequencing (NGS) has made microbiome studies more accessible to a wider audience of researchers through increases in throughput and falling costs (Schwarze et al., 2020). Common remaining bottlenecks for larger‐scale environmental microbiome studies are the price and the hands‐on time required for NGS‐quality genomic DNA (gDNA) extraction and NGS library preparation. Studies sampling inhibitor‐rich materials such as soil (Bahram et al., 2018; Walters et al., 2018) are further restricted to the use of specialist commercial kits (costing up to $10.26 per extraction; Marotz et al., 2017). This cost, coupled with the many handling steps, can limit studies to smaller sample numbers. Custom gDNA extraction workflows have been described, but many current methods are low in extraction yield, throughput and often not tested for NGS or microbiome purposes or optimized for soil (Abdel‐Latif & Osman, 2017; Zou et al., 2017). Commonly used protocols for nucleic acid purification are often column and centrifuge based, which are more laborious, harder to automate and so not easily used in a high‐throughput manner or scaled economically (Hamedi et al., 2016; Miao et al., 2014; Narayan et al., 2016; Oberacker et al., 2019). More recent open‐source protocols for rapid DNA purification using coated magnetic particles (Oberacker et al., 2019) or even cellulose (Zou et al., 2017) address many of these shortcomings. Carboxylated or silica‐coated magnetic particles as described by Oberacker et al. (2019) are commonly used in most NGS laboratories and can be readily automated, thus driving down costs effectively (Fisher et al., 2011). Researchers face a similar bottleneck with Illumina amplicon library constructions for microbiome typing (e.g., using 16S, ITS or 18S markers) as for nucleic acid extraction. Commercial kits are limited to a small number of barcoded libraries (Minich, Humphrey, et al., 2018), while specialist workflows (e.g., the Earth Microbiome Project benchmarked protocols) use custom sequencing primers and therefore cannot be processed using standard Illumina protocols. This limits the choice of the available sequencing provider and affects throughput and sequencing prices (Walters et al., 2016) restricting many projects to lower throughput in‐house platforms such as the Illumina MiSeq. For comparison, the Illumina MiSeq v2 500‐cycle kit has an 8.5 Gb maximal output whereas the NovaSeq 6000 SP 500‐cycle kit has a 400 Gb maximal output (Bahram et al., 2018; Bartoli et al., 2008; Thiergart et al., 2020).

**FIGURE 1:**
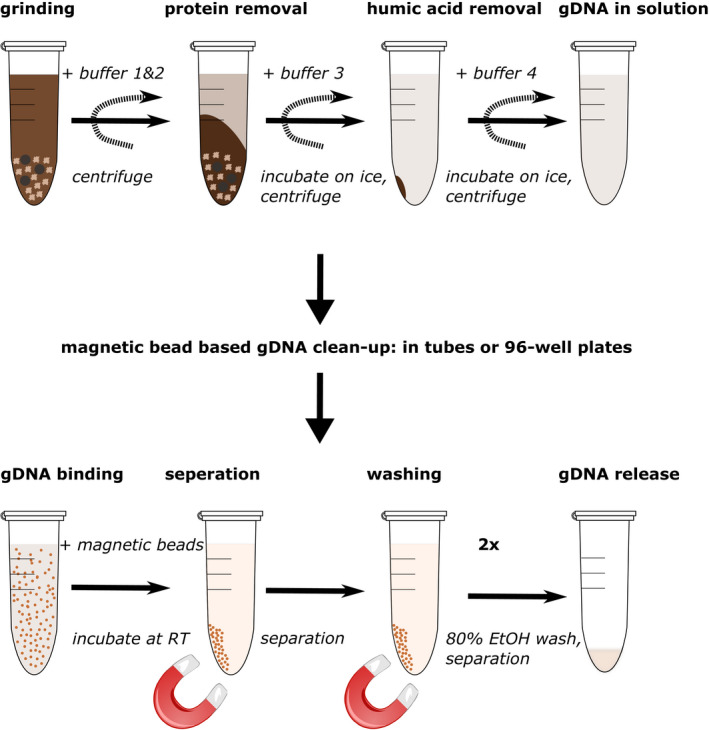
Overview of the SDE soil gDNA extraction protocol: The genomic DNA extraction protocol is divided into two parts, the gDNA extraction and the gDNA cleanup. The soil material is ground with buffer one and two, following centrifugation the proteins are removed with buffer three and incubation on ice. After centrifugation, humic acid is removed by using buffer four and incubation on ice. The extracted genomic DNA is then ready for cleanup after centrifugation. The gDNA cleanup is performed using magnetic bead‐based isopropanol precipitation of the gDNA, plus two bead washing steps with 80% Ethanol. The gDNA may then be eluted into a buffer of choice—in our case TE. gDNA, genomic DNA; SDE, soil gDNA extraction method

**FIGURE A1:**
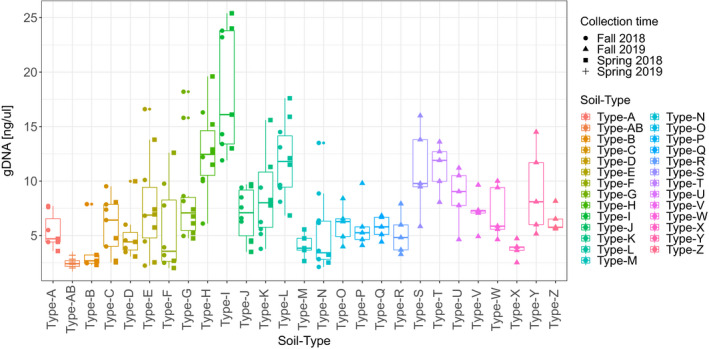
Concentration of gDNA of plate extracted gDNA from 27 different soil types of 196 samples collected throughout four seasons (spring 2018, fall 2018, spring 2019, fall 2019). gDNA, genomic DNA

In scenarios with a high number of low biomass and inhibitor‐rich samples such as rhizosphere and soil (Lakay et al., 2007; Zhou et al., 1996), it is therefore often too costly to perform large‐scale amplicon sequencing projects with a sufficient number of samples necessary for robust statistical analysis (Bahram et al., 2018; Kelly et al., 2015). To address this, we implemented a soil DNA extraction workflow (hereafter referred to as the SDE method) by combining aluminum sulfate based humic acid removal with a magnetic bead gDNA cleanup (Figure 1) and a two‐step PCR protocol creating Illumina sequencing‐ready libraries. We show equal or better gDNA extraction performance from various soil types in comparison with two commercial kits and a recently published non‐commercial extraction method by Zou et al. (2017; Figure A1). Further, we achieve this at a fraction of the cost per extraction (SDE: $0.36, MP Biomedicals™ FastDNA™ SPIN: $10.26, MoBio (now QIAGEN) PowerSoil®: $5.75). For our dual‐indexed two‐step Illumina‐compatible amplicon library preparation protocol, we calculated a total number of 2304 dual‐barcoded sequencing libraries with a minimal Hamming distance of 4 and a cost of $2.5 per library preparation. With 438 custom‐designed barcodes on each end, our protocol in principle could be easily expanded to 191,844 samples per lane, sufficient for NovaSeq scale (e.g., ~10 billion reads at 50,000 reads per sample). The combination of our two new protocols therefore allows better utilization of state‐of‐the‐art Illumina sequencing platforms to perform large‐scale amplicon‐based microbiome studies at a reduced cost.

## 2 MATERIALS AND METHODS

### 2.1 Soil material collection

We selected three different woodlands, as this captured different soil characteristics (Augusto et al., 2002; Zhou et al., 1996): a coniferous forest (52.661750, 1.095444, UK), a mixed forest (46.394474, 11.235371, Italy), and a broad‐leafed forest (46.454682, 11.301284, Italy). We sampled the soil material from the topsoil after removing the litter layer into sterile 50 ml conical tubes (Supplier Starlab (UK) Ltd, E1450‐0800) using nitrile gloves and a sterilized shovel. The sampled material was stored in a mobile refrigerator during transportation to the laboratory where the soil was stored at 4 ˚C until used for gDNA extraction. The cereal compost mix was collected in the same manner but obtained from the John Innes Horticulture facilities (Norwich, UK).

### 2.2 MoBio PowerSoil® DNA Isolation Kit

The kit was applied following the manufacturer's instructions with one alteration. The soil material was ground using the Geno/Grinder (SPEX SamplePrep 2010) for 1 min at 1750 rpm using the supplied grinding stones. The active hands‐on time without incubation and centrifugation times is 16 min per extraction (S1, https://doi.org/10.5281/zenodo.4060156).

### 2.3 MP Biomedicals™ FastDNA™ SPIN Kit for Soil

The kit was applied following the manufacturer's instructions with no alterations, using the recommended Fastprep machine (Fastprep24, MP BIO) for the grinding step. The active hands‐on time without incubation and centrifugation times is 10 min per extraction (S1, https://doi.org/10.5281/zenodo.4060156).

### 2.4 Paperdisk method

The paperdisk extraction used is based on the protocol by Zou et al. (2017). We followed the protocol as described previously (Zou et al., 2017) with one alteration: instead of using one paperdisk (Whatman qualitative filter paper, WHA1001070; Sigma‐Aldrich Co Ltd.) per extraction, in an attempt to perform three technical PCR replicates on the same extraction, we added four disks to the extraction buffer. We washed each disk using 200 µl wash buffer per disk. To determine the extracted concentration of DNA, we transferred one disk to 50 µl TE buffer (10 mM Tris–HCl pH 8.0, 1 mM EDTA) for 4 hr and measured the gDNA concentration using a Qubit 2.0 Fluorometer (Thermo Fisher Scientific) with Qubit dsDNA BR DNA assay kit reagents (Q32853; Thermo Fisher Scientific). The remaining three disks were used in the PCR reactions with one disk per reaction, as described by Zou et al. (2017).

### 2.5 SDE method

A total of 250 mg soil was transferred to a 2 ml tube containing 300 µl sterile 1 mm diameter garnet particles (Stratech Scientific Ltd, 11079110gar‐BSP, Biospec products) and three metal 4 mm bearings (grade 1000 hardened 1010 Carbon steel ball bearings, Simply Bearings). Before grinding, we added 750 µl Buffer 1 (181 mM Trisodium phosphate, 121 mM guanidinium thiocyanate, 0.22 µM sterile filtered with Sartorius UK Ltd, 16532K) and 60 µl Buffer 2 (150 mM NaCl, 4% SDS, 0.5 M Tris pH7, 0.22 µM sterile filtered) to each tube. To lyse the bacterial and fungal cells, the tubes were transferred to the Genogrinder (Spex SamplePrep, 2010) and run for 1 min at 1750 rpm. We centrifuged the tubes at 17,000 *g* for 2 min to pellet debris, and 450 µl supernatant was transferred to a new 2 ml tube, mixed with 250 µl Buffer 3 (133 mM Ammonium acetate, 0.22 µM sterile filtered), and incubated for 10 min on ice to precipitate proteins and SDS. We centrifuged the tubes at 17,000 rcf for 3 min and transferred 500 µl clear supernatant to a new 2 ml tube. The supernatant was mixed with 200 µl Buffer 4 (60 mM aluminum sulfate, 0.22 µM sterile filtered), and the reaction incubated for 10 min on ice to enable additional protein precipitation and humic acid removal. Aluminum sulfate has previously been shown to be effective for humic acid (an enzymatic inhibitor) removal of soil material (Dong et al., 2006). We centrifuged the reaction at 17,000 rcf for 10 min and transferred either 600 µl supernatant to a 1.5 ml tube or 140 µl supernatant to a 96‐well plate. Supernatants were stored at −20°C until further use. Buffer composition for Buffer 1, 2, 3, and 4 adapted from Brolaski et al. (2008).

### 2.6 SDE single tube gDNA cleanup

To perform gDNA cleanups in single tubes, we prepared 10 µl magnetic beads (Sera‐Mag Carboxylate‐Modified Magnetic Particles (Hydrophophylic), 24152105050250, GE Healthcare Life Sciences) per extraction. The beads were transferred to a 2 ml tube and washed twice with a large volume of in 1% Tween‐20. After washing, we resuspended the 10 µl beads in 20 µl 1% Tween‐20 and added 20 µl of the Tween‐20/bead mixture to each extraction (a final Tween‐20 concentration of ~0.02%). DNA was precipitated onto the beads for 5–10 min by adding 0.7× volume of Isopropanol (420 µl). We washed the magnetic beads twice with 500 µl 80% ethanol on a magnet rack, air‐dried the beads, and eluted the gDNA in 50 µl 1× TE buffer (10 mM Tris–HCl pH 8.0, 1 mM EDTA) for 5–10 min. The eluted gDNA was transferred to a 1.5 ml tube and stored at −20°C until further use.

The active hands‐on time without centrifugation and incubation times is 8 min per extraction (S1, https://doi.org/10.5281/zenodo.4060156).

### 2.7 SDE 96‐well plate gDNA cleanup

To perform the gDNA cleanups in a 96‐well format, we transferred 140 µl supernatant to a 96‐well plate (96 Well Non‐Skirted PCR Plate, CLEAR, 4TI‐0750_50, 4titude Ltd). For each extraction, we washed 5 µl magnetic beads and eluted the beads in 5 µl 1% Tween‐20 (for 96 samples, we therefore prepared 480 µl magnetic beads). We added 5 µl of the 1% Tween‐20/bead mixture to each reaction and precipitated the DNA on the beads adding 0.7× volume of Isopropanol (98 µl). We mixed the reactions by vortexing for 5 s and incubated the plates for 5 min to precipitate the gDNA. We then washed the beads twice on a magnet rack with 100 µl 80% ethanol, removed the remaining ethanol well, and eluted the gDNA in 50 µl 1× TE buffer for 10 min. The cleaned gDNA was transferred to a fresh 96‐well plate (96 Well Non‐Skirted PCR Plate, CLEAR, 4TI‐0750_50, 4titude Ltd) and stored at −20°C until further use.

### 2.8 Genomic soil DNA extraction and quality control

We performed the gDNA extraction using three replicates per soil sample and extraction method, using 250 mg soil from the same sample for each extraction. We tested four different extraction methods: (a) the PowerSoil® DNA Isolation Kit (12888‐50; CAMBIO, now 12888‐100, DNeasy PowerSoil, QIAGEN Ltd); (b) the MP Biomedicals™ FastDNA™ SPIN Kit for Soil (11492400; Fisher Scientific); (c) our SDE method; and (d) the recently published paperdisk method (Zou et al., 2017).

We determined the gDNA concentrations (Table 1) using the Qubit 2.0 Fluorometer (Thermo Fisher Scientific) dsDNA BR DNA assay kit (Q32853; Thermo Fisher Scientific) and assessed the purity of the extracted gDNA by measuring the 260:280 and 260:230 absorbance ratios using the NanoDrop ND‐1000 Spectrophotometer (Table 1). To assess the fragment size of the extracted gDNA, we used the 2200 TapeStation (Agilent Technologies) genomic DNA screen tape (5067‐5365, Agilent Technologies) and genomic DNA reagents (5067‐5366; Agilent Technologies).

**TABLE 1:** Genomic DNA quality control: Qubit BR DNA kit was used to measure the total gDNA concentration, and the nanodrop was used to compare the A260/280 and A260/230 ratios. The MoBio PowerSoil® kit delivers high quality but low yields whereas the MP Biomedicals™ FastDNA™ SPIN kit delivers high yields at low quality. The SDE method falls between the two commercial kits for both quality and quantity

Soil type	MoBio						MP						SDE				
	[ng]		A260/280		A260/230		[ng]		A260/280		A260/230		[ng]		A260/280		A260/230
Cer	0 ± 0	−8.77 ± 18.65		0.82 ± 0.3		1914.67 ± 57.87		1.75 ± 0.07		0.06 ± 0.01		468 ± 26.46		1.31 ± 0.05		0.74 ± 0.02	
MiF	1203.33 ± 540.12	2.04 ± 0.19		1.77 ± 0.46		20333.33 ± 3395.13		1.87 ± 0.02		0.26 ± 0.04		2913.33 ± 451.81		1.45 ± 0.07		0.86 ± 0.1	
BrF	1036 ± 699.6	2.12 ± 0.09		1.68 ± 0.3		8400 ± 1805.33		1.82 ± 0.06		0.16 ± 0.04		1810 ± 671.79		1.33 ± 0.02		0.87 ± 0.02	
CoF	73.33 ± 127.02	2.02 ± 0.59		0.90 ± 0.38		6920 ± 682.35		1.26 ± 0.4		0.35 ± 0.28		942.67 ± 241.17		1.29 ± 0.01		0.60 ± 0.01	

Abbreviations: BrF, broad‐leafed forest; Cer, John Innes cereal compost mix; CoF, coniferous forest; MiF, mixed forest.

### 2.9 Amplicon library construction, quality control, and pooling

We targeted the bacterial variable (V4) region of the 16S rRNA gene and the fungal ITS1 region of the internal transcribed spacer (between 18S and 5.8S rRNA subunit) for amplicon library construction. Amplicon library construction was performed using a two‐step PCR protocol: In a first PCR, we targeted the 16S V4 and ITS regions using gene‐specific primers with a 5ʹ primer tail that allows the addition of the barcoded sequencing adapters for custom dual indexing in a second PCR (Giolai et al., 2019; Rowan et al., 2019; S2, https://doi.org/10.5281/zenodo.4060156). For the bacterial 16S V4 PCRs, we included PNAs (peptide nucleic acid PCR clamps) targeting plant mitochondria (mPNA) and chloroplast regions (cPNA; Lundberg et al., 2013) in our first PCR to minimize plant gene amplification.

For the first PCR, we used the following primers adapted from Walters et al. (2016): 16S 515 forward 5ʹ‐[TCGTCGGCAGCGTC][AGATGTGTATAAGAGACAG][GT][GTGYCAGCMGCCGCGGTAA]‐3ʹ (5ʹ‐[P5][Tn5 adapter][linker][16SV4]‐3ʹ), 16S 806 reverse 5ʹ‐[GTCTCGTGGGCTCGG][AGATGTGTATAAGAGACAG][CC][GGACTACNVGGGTWTCTAAT]‐3ʹ (5ʹ‐[P7][Tn5 adapter][linker][16SV4]‐3ʹ), ITS1 forward 5ʹ‐ [TCGTCGGCAGCGTC][AGATGTGTATAAGAGACAG][GG][CTTGGTCATTTAGAGGAAGTAA]‐3ʹ (5ʹ‐[P5][Tn5 adapter][linker][ITS]‐3ʹ), and ITS2 reverse 5ʹ‐[GTCTCGTGGGCTCGG][AGATGTGTATAAGAGACAG][CG][GCTGCGTTCTTCATCGATGC]‐3ʹ (5ʹ‐[P7][Tn5 adapter][linker][ITS]‐3ʹ). All primers were ordered from Integrated DNA Technologies (IDT). The 5ʹ tails of the gene 16S V4 and ITS specific primers contain the Illumina Nextera Tn5 transposase adapter and linker sequences. This allows further amplification of the amplicons with barcoded Illumina adapters and sequencing using Illumina chemistry with the Illumina supplied sequencing or indexing primers (all oligonucleotide sequences are in S2, https://doi.org/10.5281/zenodo.4060156).

We performed the first PCR step using 3 ng gDNA input, 1 Unit Kapa HiFi polymerase (KK2102; Roche), 1× Kapa HiFi Fidelity buffer (KK2102; Roche), 0.25 µM reverse and 0.25 µM forward primer (IDT), 1× KAPA Enhancer 1 (KK5024; Sigma‐Aldrich Co Ltd), 0.3 µM dNTPs (KK2102; Roche), 1.25 µM cPNA (PNA BIO INC), 1 µM mPNA (PNA BIO INC), and DNase/RNase‐free distilled water (10977‐049; Thermo Fisher Scientific) in 10 µl total reaction volume. We performed each PCR with three technical replicates using the following cycle conditions: initial denaturation at 95°C for 3 min, followed by 20 cycles of denaturation at 98°C for 20 s, PNA clamping at 75°C for 10 s, primer annealing at 55°C for 30 s, elongation at 72°C for 30 s, with a final elongation step at 72°C for 3 min (Alpha Cycler 4, PCRmax; Labtech International Ltd). The PCR mix for the fungal libraries was the same but with ITS instead of 16S V4 primers and without the PNA oligo blockers. ITS amplification reactions were run with the following cycle conditions: initial denaturation at 95°C for 3 min, followed by 20 cycles of denaturation at 98°C for 20 s, annealing at 55°C for 30 s, elongation at 72°C for 30 s, with a final elongation at 72°C for 3 min (Alpha Cycler 4, PCRmax; Labtech International Ltd.).

After this first gene targeting PCR step, we pooled the three technical replicates reactions for the same sample and conducted a 0.7× magnetic bead cleanup (HighPrep^TM^ PCR Clean‐up System, AC‐60050, MAGBIO). The clean PCR products were eluted in 10 µl 1× TE buffer. A second PCR was conducted on the pooled replicate sample with the same program for bacterial and fungal libraries, that is, using 1× Kapa HiFi Fidelity buffer (KK2102; Roche), 1 Unit Kapa HiFi polymerase (KK2102; Roche), 0.2 µM P5 indexing primer (IDT), 0.2 µM P7 indexing primer (IDT), 0.3 µM dNTPs (KK2102; Roche), and 7.6 µl of clean gene targeting PCR product and DNase/RNase‐free distilled water (10977‐049; Thermo Fisher Scientific) in a total reaction volume of 30 µl. The barcoding cycle conditions (Alpha Cycler 4, PCRmax; Labtech International Ltd.) were as follows: initial denaturation at 95°C for 3 min, followed by 15 cycles of denaturation at 98°C for 20 s, annealing at 62°C for 30 s, elongation at 72°C for 30 s, with a final elongation at 72°C for 3 min. After the barcoding PCR step, the reactions were cleaned using a 0.7× magnetic bead cleanup (HighPrep^TM^ PCR Clean‐up System, AC‐60050, MAGBIO) and the final libraries eluted in 20 µl EB buffer (10 mM Tris–HCl pH 8.5).

We quantified the cleaned amplicon libraries using the Qubit 2.0 Fluorometer (Thermo Fisher Scientific) with dsDNA HS Assay Kit reagents (Q32854; Thermo Fisher Scientific) and controlled the size of the amplicons on the GX Touch using the 3 K kit (X‐Mark DNA LabChip, CLS144006, HT DNA NGS 3 K Reagent Kit, Perkin Elmer LAS (UK) LTD). The amplicons were pooled equimolarly to 1.5 nM for bacterial and 2.0 nM for fungal libraries according to the molarity obtained by the LabChip GX Touch smear analysis of the region between 380 and 650 bp. We performed a final 0.7× magnetic bead cleanup (HighPrep^TM^ PCR Clean‐up System, AC‐60050, MAGBIO) of the libraries and eluted the final library pools in 50 µl EB buffer.

### 2.10 Sequencing

The 16S and ITS library pools were sequenced using the MiSeq Nano reagent version 2, 500 cycle kit (Illumina) at an 8 pM loading concentration with a 10% PhiX spike‐in. 16S and ITS pools were sequenced separately—each pool using a MiSeq Nano reagent kit.

### 2.11 Amplicon data analysis

We demultiplexed bcl files using bcl2fastq version 2‐2.20.0.422 with the settings—barcode‐mismatches 1—fastq‐compression‐level 9 into individual fastq.gz files. We trimmed the paired‐end reads for primers, sequencing adapters and linker sequences using cutadapt‐1.9.1 (Martin, 2011) with the settings ‐n 4—minimum‐length = 50. The data were quality controlled using R‐3.5.0 and DADA2 version 1.8.0 according to the workflow described in (Callahan et al.,) version 2. The truncation length for forward reads was set to 180 bp and the truncation length for the reverse reads to 200 bp. For 16S libraries, we used the following parameters: maxN = 0, maxEE = 2 and truncQ = 11. For ITS libraries, we specified the following parameters: maxN = 0, maxEE = c(2, 2) and truncQ = 11 and a minimum length of 50 bp. Forward and reverse reads were merged with default settings. We used the Silva (silva_nr_v132) database to classify bacterial reads (Quast et al., 2013) and UNITE (sh_general_release_dynamic_s_01.12.2017) for the fungal dataset (Nilsson et al., 2019). Reads that did not match to the bacterial or fungal database were removed. Alpha‐diversity analysis (Shannon and Observed measure) was performed on pre‐normalized data (package "phyloseq," R‐3.5.0, version 2.5.2). For beta‐diversity analysis, ASVs with a mean lower as 10^−5^ were removed from the datasets. The filtered data with 4113 bacterial ASVs and 1602 fungal ASVs (package "phyloseq," version 1.24.0) were used to calculate the β‐diversity (Bray–Curtis, R‐3.5.0 "vegan" package, version 2.5.2) and to perform statistical analysis (package "vegan," ANOSIM and PERMANOVA: adonis function; Dixon, 2003). All numbers of processed reads through the analysis pipeline are in S3, https://doi.org/10.5281/zenodo.4060156.

We performed the correlation analysis in R‐3.5.0 using the filtered phyloseq object on genus level and plotted it with log10 scaling (McMurdie & Holmes, 2013). The corrplot was generated in R, using the filtered phyloseq object on order level with the corrplot package version 0.84. All figures were generated in R‐3.5.0 using the R package ggplot2‐3.1.1 (Ginestet, 2011).

## 3 RESULTS

### 3.1 DNA yield and fragment analysis of different extraction methods

**FIGURE A2:**
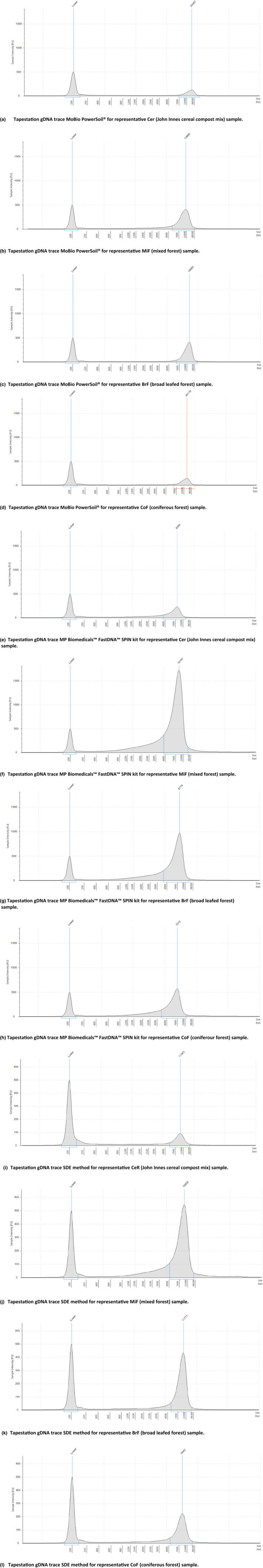
Genomic TapeStation traces of representative MoBio PowerSoil®, MP Biomedicals™ FastDNA™ SPIN and SDE gDNA extractions. gDNA, genomic DNA; SDE, soil gDNA extraction method

We tested our SDE method by extracting gDNA from 250 mg samples of four different soil types taken from a mixed forest (MiF), a coniferous forest (CoF), and a broad‐leafed forest (BrF), plus a standardized cereal crop compost mix used at the John Innes Centre (Cer). We compared our method to two frequently used commercial extraction kits: MP Biomedicals™ FastDNA™ SPIN and MoBio PowerSoil® and a recently published low‐cost paperdisk method described to extract microbial DNA suitable for PCR in less than 30 s (Zou et al., 2017). We first determined which gDNA extraction method produces the highest yield and best gDNA quality using fluorometric and spectrophotometric analysis. The MP Biomedicals™ FastDNA™ SPIN kit delivered the highest and the MoBio PowerSoil® kit the lowest gDNA yield (Table 1). The highest gDNA purity (A260/A280 and A260/A230 ratios) was obtained by the MoBio PowerSoil® kit and the lowest by the MP Biomedicals™ FastDNA™ SPIN kit (Table 1). Our method scored between the two commercial kits for both quality and quantity (Table 1). We further evaluated the methods for the extracted gDNA fragment length on the Agilent TapeStation. For the MoBio PowerSoil® kit method, we found the majority of gDNA fragment sizes fall between 13.9 and 24.4 kb. The SDE method produced fragments centered between 11.3 and 11.7 kb and the MP Biomedicals™ FastDNA™ SPIN mostly extracted fragments below 10 kb (Figure A2). For the paperdisk method, we could not obtain enough DNA for fragment analysis.

### 3.2 Extraction method effects on bacterial and fungal amplicon library construction

**FIGURE 2:**
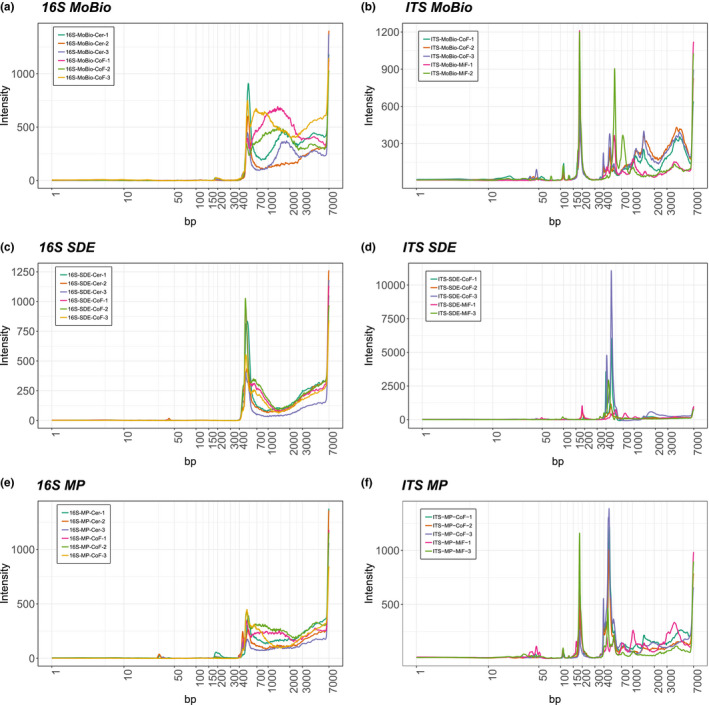
Representative results comparing MoBio PowerSoil®, MP Biomedicals™ FastDNA™ SPIN and SDE methods run on a LabChip GX Touch: (a) Representative 16S libraries of gDNA extracted with MoBio PowerSoil®. (b) Representative ITS libraries of gDNA extracted with MoBio PowerSoil®. (c) Representative 16S libraries of gDNA extracted with SDE. (d) Representative ITS libraries of gDNA extracted with SDE. (e) Representative 16S libraries of extracted gDNA with MP Biomedicals™ FastDNA™ SPIN. (f) Representative ITS libraries of extracted gDNA with MP Biomedicals™ FastDNA™ SPIN. The target size of representative 16S libraries is between 350 and 500 bp and for ITS libraries between 300 and 700 bp. gDNA, genomic DNA; SDE, soil gDNA extraction method [Correction added on 11 Dec 2020, after first online publication: Figure 2 has been updated to correct minor inaccuracies.]

We constructed 16S V4 and ITS rRNA Illumina sequencing libraries from all extractions (three biological replicates per soil type) using 3 ng of gDNA input per library construction reaction and three technical replicates (similar to Tourlousse et al., 2018). All libraries were inspected using LabChip GX Touch high‐sensitivity capillary electrophoresis. The gDNA extracted with MP Biomedicals™ FastDNA™ SPIN, MoBio PowerSoil®, and our SDE method performed well in library construction, producing libraries with similar profiles (Figure 2). The paperdisk method did not produce a library with a detectable electropherogram trace. We pooled all libraries at equal mass (except the paperdisk method where we used the full amount as these libraries were not detectable) and submitted each library for 250 bp paired‐end sequencing. Sequencing of the paperdisk extraction method did not produce any reads suggesting that the extracted gDNA concentration was too low for successful library construction.

### 3.3 Comparison of extraction methods based on bacterial and fungal microbial composition

**FIGURE 3:**
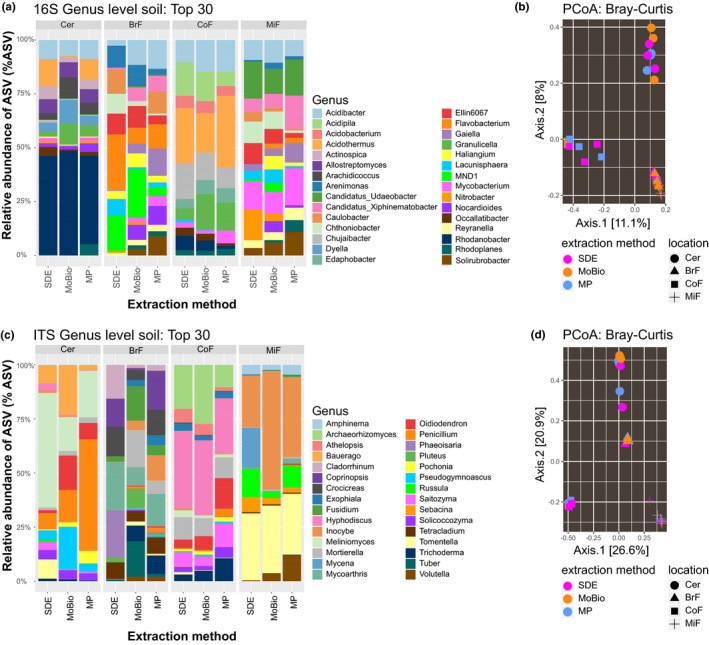
PCoA comparison of ASV abundances and relative abundance bar chart for bacterial and fungal communities of three different gDNA extraction methods and four soil types: (a) Top 30 bacterial community composition representing 0.72% of the overall bacterial community. (b) PCoA showing beta diversity by Bray–Curtis distance for bacterial community composition. (c) Top 30 fungal community composition representing 1.87% of the overall fungal community. (d) PCoA showing beta diversity by Bray–Curtis distance for fungal community composition. (a) and (b) show that the clustering for the entire bacterial communities is soil type‐dependent and not driven by different gDNA extraction methods. (c) and (d) show the same is true for the fungal community structure. Statistical analysis shows no significant difference between DNA extraction methods used for both bacterial (adonis test, *p*‐value: .579) and fungal (adonis test, *p*‐value: .801) communities, but shows a significant difference between locations for bacterial (ANOSIM test, *p*‐value: 9.99e–4) and fungal (ANOSIM test, *p*‐value: 9.99e‐4) communities. ASVs, amplicon sequence variants; BrF, broad‐leafed forest soil; Cer, standard cereal compost used at the JIC; CoF, coniferous forest soil; MiF, mixed forest soil; SDE, soil gDNA extraction method, MoBio, MoBio PowerSoil®; MP, MP Biomedicals™ FastDNA™ SPIN

**FIGURE 4:**
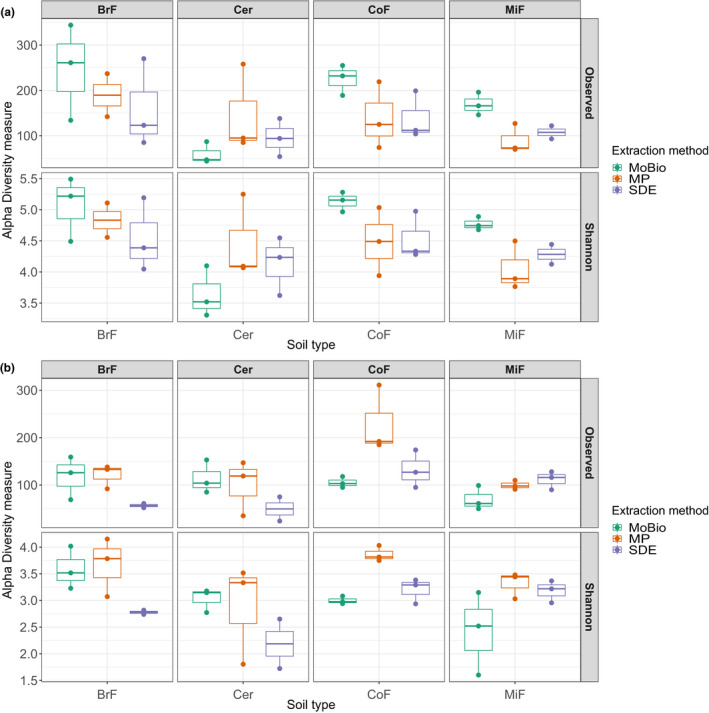
Alpha‐diversity comparison for bacterial and fungal communities of three different gDNA extraction methods and four soil types: (a) Bacterial alpha‐diversity overview of the observed richness and Shannon diversity. No influence on alpha diversity by extraction method (Shannon diversity ANOVA test, *p*‐value: .466618, observed richness Kruskal test, *p*‐value: .36554), but by soil type (Shannon diversity ANOVA test, *p*‐value: .018507, Observed richness Kruskal test, *p*‐value: .032519). No influence by soil type on fungal alpha diversity (Shannon diversity Kruskal test, *p*‐value: .21473, observed diversity Kruskal test, *p*‐value: .103862) and some influence of extraction method (Shannon diversity Kruskal test, *p*‐value: .018628, observed richness Kruskal test, *p*‐value: .168873). BrF, broad‐leafed forest soil; Cer, standard cereal compost used at the JIC; CoF, coniferous forest soil; gDNA, genomic DNA; MiF, mixed forest soil; MoBio, MoBio PowerSoil®; MP, MP Biomedicals™ FastDNA™ SPIN; SDE, soil gDNA extraction method

**FIGURE 5:**
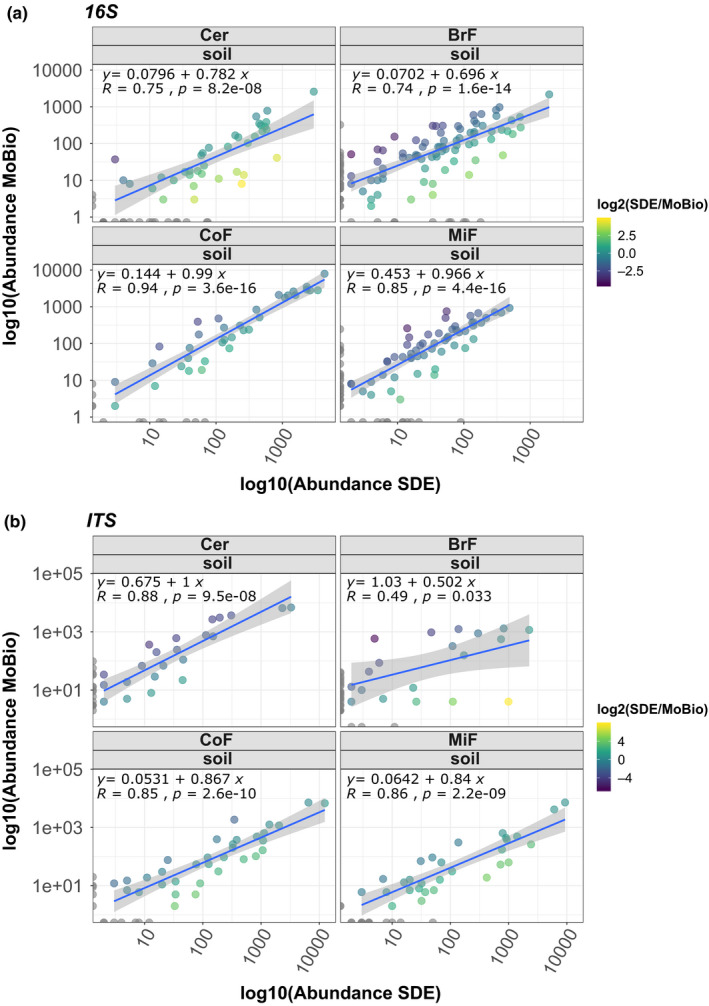
Correlation analysis of bacterial and fungal ASVs from SDE versus MoBio PowerSoil® gDNA extraction: (a) shows bacterial ASV correlation between MoBio PowerSoil® and SDE for four different soil types. (b) shows fungal ASV correlation between MoBio PowerSoil® and SDE for four different soil types. Both extraction methods led to bacterial and fungal ASV that show a positive correlation, indicating that there is no extraction method‐induced bias regarding sequenced ASV. Abundances on *x* and *y* axes were log10 scaled, each dot represents a genus, and dots are colored as log2(Abundance[SDE]/Abundance[Commercial Kit]). Gray dots on each axis are Genera uniquely detected for the extraction method. ASV, amplicon sequence variants; BrF, soil from a broad‐leafed forest; Cer, standard cereal crop soil mix used at JIC; CoF, soil from a coniferous forest; MiF, soil from a mixed forest

**FIGURE A3:**
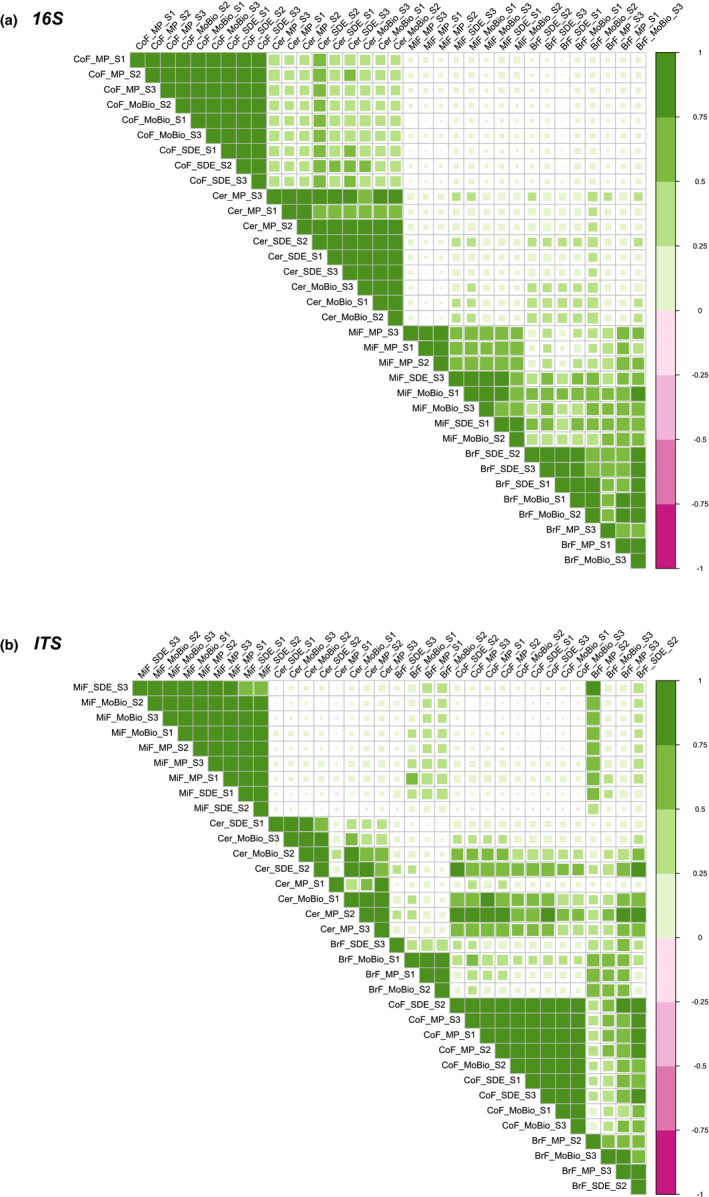
Corrplot of technical replicates of MP Biomedicals™ FastDNA™ SPIN, MoBio PowerSoil® and SDE method. Order level. SDE, soil gDNA extraction method

**FIGURE A4:**
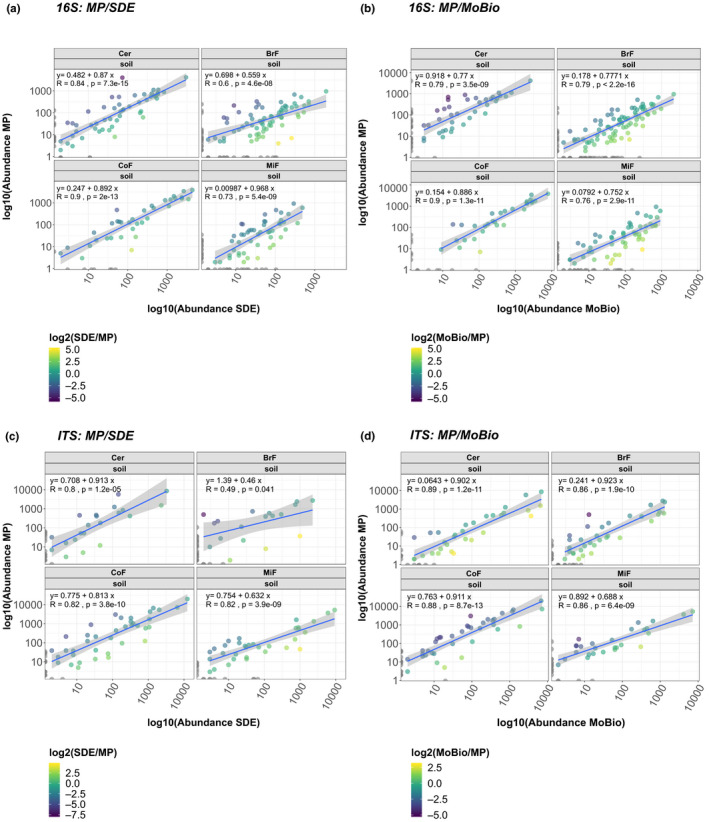
Correlation analysis SDE method versus MP Biomedicals™ FastDNA™ SPIN & MP Biomedicals™ FastDNA™ SPIN versus MoBio PowerSoil®(16S/ITS). SDE, soil gDNA extraction method

It has been previously reported that different microbial gDNA extraction methods can introduce a genera bias (Sáenz et al., 2019). To test this, we compared the biological replicates of each library preparation method (apart from the paperdisk method) by correlation analysis of the detected bacterial (total of 4069) and fungal (total of 1549) amplicon sequence variants (ASVs; Table 2, Figure A3). The three tested gDNA extraction methods compared well across all soil types. We also compared the genus abundances of our SDE method with the two commercial kits by analyzing the bacterial and fungal genus abundances of each library. The genus abundance plots for each soil type were not statistically significantly different between the extraction methods used for either fungal (adonis test, *p*‐value: .801, Table 3) or bacterial communities (adonis test, p‐value: 0.579, Table 3). Instead, our data showed statistically significant variation between soil types (bacteria ANOSIM test, *p*‐value: 9.99e–4, fungi ANOSIM test, p‐value: 9.99e‐4, Table 3) but not between gDNA extraction methods (bacteria: Figure 3a,b and fungi: Figure 3c,d). We further tested the samples using beta diversity as a measure (Bray–Curtis) for between‐sample similarity. This analysis agreed with the result of the genus abundance plots, that is, by clustering the soil types separately but not clustering the data for the three extraction methods (Figure 3b,d). We confirmed this result with permutation multivariate analysis of variance analyses (PERMANOVA, package "vegan" version 2.5.2, adonis function). We also controlled the impact of gDNA extraction methods on alpha diversity (Figure 4). We could not find an impact of the gDNA extraction method on the bacterial alpha diversity (Shannon diversity ANOVA test, p‐value: 0.466618, Observed richness Kruskal test, p‐value: 0.36554), but observed that bacterial alpha‐diversity differences are driven by soil type (Table 3, Shannon diversity ANOVA test, p‐value: 0.018507, Observed richness Kruskal test, p‐value: 0.032519). Fungal alpha diversity does not show a significantly different effect due to soil type (Table 3, Shannon diversity Kruskal test, *p*‐value: .21473, Observed richness Kruskal test, *p*‐value: .103862) and only minor differences of the gDNA extraction method (Shannon diversity Kruskal test, *p*‐value: .018628, Observed richness Kruskal test, *p*‐value: .168873). To compare the extraction methods in more detail and study any potential ASV‐related bias, we compared the ASV abundances of each kit with the abundances assessed with our method. In the SDE to MoBio PowerSoil® kit comparison, we found the following correlation coefficients for bacterial ASVs over the different soil types: Cer 0.75, BrF 0.74, CoF 0.94, MiF 0.85 (Figure 5a; Table 2) and for fungal ASVs: Cer 0.88, BrF 0.49, CoF 0.85, MiF 0.86 (Figure 5b; Table 2). The correlation analysis of the SDE to MP Biomedicals™ FastDNA™ SPIN kit delivered similar results (Cer 0.84, BrF 0.6, CoF 0.9, MiF 0.73 for bacteria, Figure A4a and Cer 0.8, BrF 0.49, CoF 0.82, MiF 0.82 for fungi, Figure A4c; Table 2). These results confirm that our SDE method fits between two commonly used commercial soil gDNA extraction kits across a broad range of measurable parameters.

**TABLE 2:** Correlation coefficient between different extraction methods and on four different soil types for fungal and bacterial ASVs: Correlation coefficient between different gDNA extraction method derived ASVs for all extraction method combinations for fungal and bacterial data (SDE/MoBio, SDE/MP, and MoBio/MP)

	Soil type	Extraction method	*R*
16S	Cer	SDE/MoBio	.75
16S	BrF	SDE/MoBio	.74
16S	CoF	SDE/MoBio	.94
16S	MiF	SDE/MoBio	.85
16S	Cer	MP/SDE	.84
16S	BrF	MP/SDE	.6
16S	CoF	MP/SDE	.9
16S	MiF	MP/SDE	.73
16S	Cer	MP/MoBio	.79
16S	BrF	MP/MoBio	.79
16S	CoF	MP/MoBio	.9
16S	MiF	MP/MoBio	.76
ITS	Cer	SDE/MoBio	.88
ITS	BrF	SDE/MoBio	.49
ITS	CoF	SDE/MoBio	.85
ITS	MiF	SDE/MoBio	.86
ITS	Cer	MP/SDE	.8
ITS	BrF	MP/SDE	.49
ITS	CoF	MP/SDE	.82
ITS	MiF	MP/SDE	.82
ITS	Cer	MP/MoBio	.89
ITS	BrF	MP/MoBio	.86
ITS	CoF	MP/MoBio	.88
ITS	MiF	MP/MoBio	.86

Abbreviations: BrF, soil from a broad‐leafed forest; Cer, standard cereal crop soil mix used at JIC, Norwich, UK; CoF, soil from a coniferous forest; MiF, soil from a mixed forest.

**TABLE 3:** Statistical results of alpha‐ and beta‐diversity analysis: Bacterial and fungal data were investigated for potential soil type and gDNA extraction method influence on alpha and beta diversity. For alpha diversity, Shannon diversity and Observed richness were used, and for beta diversity, Bray–Curtis distance matrix was used.

	Question	Alpha diversity			Beta diversity		
		Measure	Kruskal [*p*‐value]	ANOVA	Distance	ANOSIM	Adonis
16S	Soil type	Shannon		0.018507038			
16S	Soil type	Observed	0.032518656				
16S	Extraction method	Shannon		0.466618041			
16S	Extraction method	Observed	0.365539882				
16S	Soil type				Bray–Curtis	0.000999001	
16S	Extraction method				Bray–Curtis		0.579
ITS	Soil type	Observed	0.103862				
ITS	Soil type	Shannon	0.214273				
ITS	Soil type				Bray–Curtis	0.000999001	
ITS	Extraction method	Shannon	0.018627631				
ITS	Extraction method	Observed	0.168872594				
ITS	Extraction method				Bray–Curtis		0.801

## 4 DISCUSSION

Custom gDNA extraction methods for soil samples have been described previously (Bürgmann et al., 2001; Fatima et al., 2014; Robe et al., 2003; Verma et al., 2017; Yeates et al., 1998; Zhou et al., 1996). These methods emphasized gDNA quantity (Bürgmann et al., 2001; Fatima et al., 2014), quality (Bürgmann et al., 2001; Fatima et al., 2014; Verma et al., 2017), or cost‐efficiency (Devi et al., 2015; Fatima et al., 2014; Yeates et al., 1998; Zou et al., 2017). However, an often overlooked but practically important consideration is the hands‐on time required per extraction without a quantity or quality penalty. KatharoSeq (Minich, Zhu, et al., 2018), for example, is a pipeline for low biomass samples that delivers good gDNA quality with less hands‐on time; however, it still uses parts of a commercial kit, which increases the price per sample. On the other hand, Zou et al. described a fast and very affordable gDNA extraction method but yielding lower gDNA quantities (Zou et al., 2017). Here, we present a high‐throughput gDNA extraction method that is suitable for low input and inhibitor‐rich sample types such as soils (Figure 1; Figure A1). For our method, we first optimized mechanical lysis conditions by increasing the amount and types of grinding material, then chemical additives to the lysis buffer which remove common contaminants. We also compared commercial kits (silica column and carboxylate‐coated beads) with our carboxylated magnetic bead‐based protocol to determine best extraction yields. For the additives, we used sodium phosphate as a buffer matrix, which in the past together with aluminum ions was recommended for efficient removal of humic acids while minimizing DNA losses during extraction (Mandalakis et al., 2018). We found that adding aluminum sulfate in the lysis buffer leads to increased DNA yields and purity. Although we successfully extracted DNA adding only aluminum sulfate, we also observed that the addition of ammonium acetate to further precipitate impurities (Yu & Mohn, 1999) increased PCR amplification success (S4, https://doi.org/10.5281/zenodo.4060156 for more experimental details). We compared our finalized protocol to two leading commercial (MoBio PowerSoil® and MP Biomedicals™ FastDNA™ SPIN kits) and a non‐commercial (paperdisk based) extraction method (Zou et al., 2017) and observed that our SDE method delivered good quality and quantities of NGS‐compatible gDNA at a fraction of the cost of commercially available solutions. We also find that our method requires less manually intensive centrifugation steps (MoBio: nine steps, MP: nine steps, SDE: three steps) and the total hands‐on time of our method is lower than the hands‐on times for the MoBio and MP kits (MoBio: 16 min, MP: 10 min, SDE: 8 min). We achieved this with the use of a modified SPRI bead extraction protocol that allows fast, scalable, and inexpensive extraction of nucleic acids (Oberacker et al., 2019), especially because magnetic particles enable the transfer of our protocol to a plate format, without the disadvantages of handling many tubes and minimizing potential sample mix‐ups (S1, https://doi.org/10.5281/zenodo.4060156). We tested our extraction method for 96‐well plate compatibility by quantifying yields from 27 different soil types of 196 samples (Figure A1). Because it uses simple pipetting steps, SPRI bead‐based purification and washing steps, our method should be easily adaptable from a multi‐channel pipette to common liquid handling robotic systems (typically already able to use bead‐based methods for DNA and RNA NGS library construction). The extracted gDNA from four distinctively different soil types using SDE is similar in quality and quantity to the two commercial kits (Table 1), with the extracted gDNA from the commercial kits and the SDE method led to similar amplicon library profiles (Figure 2). In contrast, the paperdisk method did not generate useable sequencing libraries. We further investigated the gDNA preparation methods for extraction biases when analyzing fungal and bacterial communities. Here, the results of the two commercial kits and the SDE method overlap, showing no statistical differences in microbiome composition (Figure 3) for beta diversity. Clustering of the sequencing data using PCoA separated soil types but not gDNA extraction methods (Figure 3b,d). A correlation analysis between SDE and MoBio, SDE and MP, and MoBio and MP, for detected bacterial and fungal communities (Figure 5; Figures A3 and A4), showeda strong correlation between the commercial kits and the SDE method the one exception being the fungal BrF samples. We suspect that the lower correlation of 0.49 between SDE and MoBio and SDE and MP for the fungal BrF soil samples (Table 3) could potentially be due lower read depth of the SDE BrF samples compared to the other samples (S3, https://doi.org/10.5281/zenodo.4060156). Further, bacterial alpha‐diversity analysis for Shannon diversity index and Observed richness is not affected by gDNA extraction methods, but only by soil type (Table 3). Fungal alpha diversity is not affected by soil type and only partly affected by gDNA extraction method (Table 3; Figure 4). This altogether indicates that our SDE method overall does not induce an experimental bias in extracting bacterial and fungal community data.

## 5 CONCLUSION

To conclude, we present a low‐cost gDNA extraction method ($0.36/sample, see S5, https://doi.org/10.5281/zenodo.4060156 for detailed breakdown) that effectively extracts soil samples and delivers good quality and quantities of gDNA suitable for microbiome studies. We show that the SDE method does not introduce a library preparation and therefore a sequencing bias. We also present a low‐cost custom and fully Illumina‐compatible bacterial and fungal amplicon library construction protocol that can multiplex up to 2304 samples to one pool ($2.5/library). Our method, therefore, enables researchers to sequence their projects on any available Illumina platform without the need to purchase full lanes/flow cells. Overall, the two presented methods will enable microbiome projects to be performed at any desired scale at an affordable price for a broad audience of microbiome enthusiasts.

## ETHICS STATEMENT

None required.

## CONFLICT OF INTEREST

None declared.

## AUTHOR CONTRIBUTION

**Anita Bollmann‐Giolai:** Conceptualization (supporting); Formal analysis (lead); Investigation (lead); Methodology (supporting); Writing‐original draft (lead); Writing‐review & editing (supporting). **Michael Giolai:** Data curation (supporting); Formal analysis (supporting); Investigation (supporting); Methodology (supporting). **Darren Heavens:** Investigation (supporting); Methodology (supporting); Resources (supporting). **Iain Macaulay:** Investigation (supporting); Methodology (supporting); Project administration (supporting); Resources (supporting). **Jacob Malone:** Conceptualization (supporting); Formal analysis (supporting); Funding acquisition (supporting); Methodology (supporting); Resources (lead); Supervision (lead). **Matthew Derek Clark:** Conceptualization (lead); Methodology (lead); Supervision (supporting); Writing‐review & editing (lead).

## Data Availability

The sequence datasets generated during the current study are available in the *European Nucleotide Archive (ENA)* repository: https://www.ebi.ac.uk/ena/browser/view/PRJEB37921. Supporting Information files are available in Zenodo: https://doi.org/10.5281/zenodo.4060156.
